# Exploring Embodied and Bioenergetic Approaches in Trauma Therapy: Observing Somatic Experience and Olfactory Memory

**DOI:** 10.3390/brainsci14040385

**Published:** 2024-04-16

**Authors:** Sara Invitto, Patrizia Moselli

**Affiliations:** 1 Laboratory on Psychophysiological and Cognitive Olfactory Processes, Department of Biological and Environmental Sciences and Technologies, University of Salento, 73100 Lecce, Italy; 2 SIAB—Società Italiana Analisi Bioenergetica, 00183 Roma, Italy; patriziamoselli1957@gmail.com; 3 IIBA International Institute for Bioenergetic Analysis, 08670 Navàs, Barcelona, Spain

**Keywords:** olfactory memory, post-traumatic stress disorder, bioenergetic analysis, embodied cognition, embodied simulation

## Abstract

Recent studies highlight how body psychotherapy is becoming highly cited, especially in connection with studies on trauma-related disorders. This review highlights the theoretical assumptions and recent points in common with embodied simulation and new sensory theories by integrating bioenergetic analysis, embodiment, and olfactory memory in trauma and post-traumatic stress disorder (PTSD) therapy. Embodied memory, rooted in sensorimotor experiences, shapes cognitive functions and emotional responses. Trauma, embodied in somatic experiences, disrupts these processes, leading to symptoms such as chronic pain and dissociation. The literature discussed highlights the impact of burning odors on individuals with PTSD and those who have experienced childhood maltreatment. Burning odors can increase stress and heart rate in war veterans, with sensitivity to these odors intensifying over time since the trauma. Additionally, adults who experienced childhood maltreatment exhibit faster processing of unpleasant odors and increased symptom severity. Grounding techniques, such as adopting a balanced posture, enhance breathing and sensory capabilities, potentially aiding in managing symptoms associated with trauma-related disorders such as PTSD.

## 1. Introduction

Recent studies highlight how body psychotherapy is becoming highly cited, especially in connection with studies on trauma-related disorders. Previously, the literature has not systematized theoretical assumptions and recent points in common with embodied simulation and new sensory theories by integrating bioenergetic analysis, embodied cognition, and olfactory memory in trauma and PTSD therapy. Trauma-related disorders (TRDs) and post-traumatic stress disorders (PTSDs) are expressions in which, by now, a somatic and psychophysiological component has clearly been highlighted. From a psychophysiological point of view, a recent meta-analysis highlighted that the anterior cingulate cortex and the bilateral amygdala are the areas most hyperactivated in PTSD [1]. In contrast, the ventromedial prefrontal cortex and the inferior frontal gyrus appear to be the most inhibited areas. The growth of PTSD involves, as a rule, the shift of the brain state from the high-level processing of multimodal contextual and mnemonic stimuli to primitive formation mediated by the amygdala, of timed sensory associations [2]. The personality differences of each individual (e.g., intelligence, neuroticism, and attention) will influence the stimulus threshold where this change occurs, and consequently affect the subject’s vulnerability [1]. These aspects, connected to an alert system, also profoundly involve the olfactory system, which shares common substrates with emotion, especially fear [3,4]. The theoretical assumptions and recent points are shared with embodied cognition and new sensory theories by integrating bioenergetic analysis, embodied simulation, and olfactory memory in PTSD therapy. Embodied memory, rooted in sensorimotor experiences, shapes cognitive functions and emotional responses. Trauma, embodied in somatic experiences, disrupts these processes, leading to symptoms such as chronic pain and dissociation. Bioenergetic analysis is body psychotherapy developed in the 1950s by Alexander Lowen [5,6] from Reichian assumptions [7]. This analysis focuses on the body’s sensory, perceptual, and cognitive expression, starting from the idea that the unconscious is not a symbolic cerebral representation, even though the unconscious is expressed through the body as a whole. The terms in bioenergetic analysis are linked to “grounding” (i.e., experience) and are strongly connected to the most recent cognitive neuroscience theories on embodied simulation and grounded cognition [8,9,10,11]. In addition, the aspects of the grounded experience link it in a very specific way to the theory of integrated information, which sees consciousness as part of a sensory process linked to causality [12,13,14].

Embodied cognition is an approach that states that mind and body are not distinct, but concur in determining mental and cognitive processes [15]. Gallagher wrote a specific cognitive contribution entitled “How the body shapes the mind”, speaking of “embodied cognition”. In bioenergetic terms, the body shapes the mind, meaning that bodily action in an ecological environment changes our cognition, emotions, and actions. Furthermore, this aspect could be taken to the extreme if we also talk about aspects of epigenetics. Bodily action can even modify the expression of our phenotypic aspects if we compare these aspects to comparative evolutionary elements. For example, recent studies on environmental (social) handling/enrichment and on neurodegenerative processes in animal models demonstrate how genotypic aspects may not manifest themselves in purely clinical phenotypic aspects [16,17]. This aspect of direct action on the body and with the body seems critical to how body therapy can be read according to different psychophysiological interpretative levels.

The concept of “embodied” begins with W. Reich and A. Lowen, and later in other somatic therapies [18,19,20]. However, simultaneously, psychophysiology and cognitive neuroscience demonstrated precisely how the body and bodily experience are essential for the development of homeostatic physiological processes and of processes connected to thought and consciousness, as are, in part, the processes described in bioenergetic terms by the bodywork [21,22].

The main concepts of bioenergetics have borrowed the main concepts of embodied theories, albeit in different ways. Grounded cognition and simulation involves the reactivation of perceptual, motor, and introspective states acquired during experience with the world, body, and mind, and offers a unifying vision of cognition [23], emphasizing dynamic brain–body–environment interactions and perception–action links as standard foundations of simple behaviors, as well as of complex cognitive and social skills. Situated cognition is based on the hypothesis that the ecological human–environment interaction can influence individual human development [24,25].

Being “grounded” is also a central aspect of bioenergetic analysis and requires a sense of rootedness that starts from motor/bodily aspects to arrive at emotional and cognitive factors [26]. The meaning of embodied memory arises from a paradigm change that has developed over time, namely the idea that the meaning of symbols is obtained through the sensorimotor experience located in an environmental context. On these premises, many theoretical studies converge on the idea that the body represents how to model higher-level executive functions such as memory.

Starting from these models, the aim of this review would provide a focus on the intersection of bioenergetic analysis, TRD, and PTSD, particularly emphasizing the somatic and psychophysiological components involved. The content explores how trauma affects both the mind and body, leading to symptoms such as chronic pain, dissociation, and altered sensory processing, according to the theoretical underpinnings of embodied simulation and bioenergetic analysis, the first school of thought in body-focused psychotherapy.

**Figure 1:**
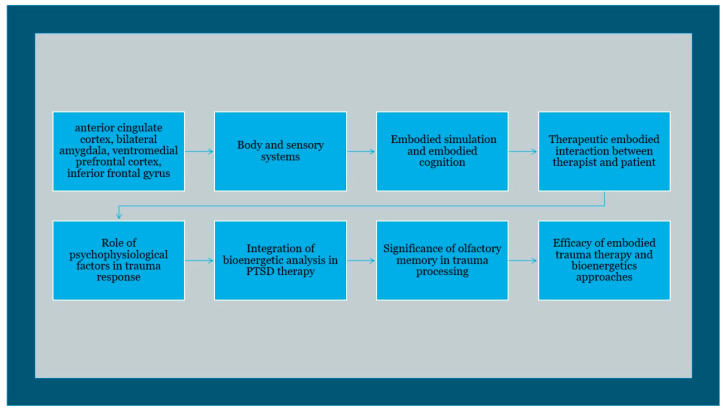
TRS and PTSD in an embodied therapeutic approach through somatic experience and olfactory memory.

Furthermore, the paper delves into the role of embodied memory, highlighting how sensorimotor experiences shape cognitive functions and emotional responses, and how trauma disrupts these processes. It explores the concept of “body muscle memory” and how trauma can lead to muscle tension and various somatic symptoms. The paper also discusses the importance of reconnecting with the body in trauma therapy, emphasizing the role of body psychotherapy in addressing trauma symptoms. Additionally, the paper examines the role of olfactory memory in trauma, emphasizing how smells can trigger traumatic memories and how olfactory stimuli are closely linked to emotional processing. It explores the potential therapeutic implications of incorporating olfactory stimuli into trauma therapy sessions. Overall, the paper aims to provide a comprehensive understanding of how trauma could affect mind–body unity and explore embodied therapeutic approaches in addressing trauma-related disorders (see Figure 1).

## 2. Embodied Memory

Common knowledge of embodied memory constitutes those memories thanks to which, for example, we remember the practical action of sitting down or that allow us to orient ourselves easily in spaces of customary use [27,28,29]. Furthermore, listening to music can viscerally evoke a conversation in the past, and hearing a particular smell can bring to mind an emotional experience [30]. Therefore, sensorimotor reactivation is configured as a constituent of the mnemonic traces through which our cognitive system can retrieve information.

Fuchs stressed that this memory is implicit, that repeated bodily experience is the basis of the process that connects the body to its intention to operate in the environment [31]. In favor of this, Merleau-Ponty expresses the idea of a sensorimotor vision of body memory, capable of providing itself with know-how or the knowledge of how to act with or towards a part of one’s body [15]. Finally, Gallese and Sinigaglia elaborate on a conception of body memory as “a multiplicity of possibilities of action that allows the practical tuning of the body with its environment” [32,33].

Through knowledge of the conceptions that have gradually been endorsed, our experience of the body is not direct, but mediated by perceptual information, influenced by internal information and recalibrated through the implicit and explicit body representation stored, which is body memory. Therefore, it is possible to idealize that memory and, in general, cognitive functions have evolved to serve human action and facilitate interactions between humans and their environment. Thus, cognitive and memory processes are grounded in human experience and intervene in a real-world environment that interferes with perception and involves action.

Individual personality is another factor that interferes with our interaction with the environment; according to modern theories, the latter develops through environmental and hereditary factors. Specific to the bodily psychotherapeutic concepts of personality, namely, the bioenergetic model, is the basic idea that life experiences and emotionally significant attitudes manifest physically. Conversely, habitual use of the body affects mental attitudes and basic moods [6]. Consequently, the body should provide relevant information about a person, not only indicating their current emotional state and/or immediate willingness to act, but also about the stable dispositions of thinking, feeling, and behaving. Therefore, according to this perspective, the body is an indispensable component of human existence, and from this point of view, the cognitive and somatic processes evolve in parallel. Another relevant concept is that memories can, to a certain extent, be triggered and brought to consciousness by affective, motor, or sensory stimuli. The experiences of interaction with the environment evoke cognitive and psychosomatic coping strategies or defense [34].

Connection with one’s body has, therefore, played a central role in psychotherapy since its inception: the founders of psychoanalytic therapy considered somatic tensions as an expression of mental conflicts. On the one hand, the body is the stage on which mental disorders develop: in primary care, most patients with psychiatric disorders have somatic symptoms. On the other hand, it is the prerequisite of the psychotherapeutic process in which the patient and therapist communicate verbally and in a bodily dialogue [35,36].

In the case of pathology, Weizsäcker’s model of the “Gestaltkreis” explains the invisibility of an individual’s psychological conflict when somatic symptoms replace it [37]. In this sense, psychotherapy strives to find an explanation and a functional solution in which thought, feeling, bodily experience, and expression can be intertwined. For example, “functional relaxation” understands breathing exercises as a semiotic process in which the therapist takes in a client’s bodily signals, interprets them as their own, and verbalizes the result. For this reason, the bioenergetic perspective fits well into the embodied mind paradigm that prevails today in the cognitive sciences. According to this position, human cognition cannot be explained only as a function of the brain, but as an interaction of the entire body and its environment. Shapiro points out that information processing begins in the sensorimotor periphery [38]. From this inevitable evolutionary perspective, human experience and behavior should be understood by their genesis and grounding. Similarly, Petzold and Sieper propose a hermeneutical point of view in which the informed body contains a person’s mental history and state [39].

Recent evidence presents the efficacy of bioenergetic psychotherapeutic treatment in an outpatient setting in Germany and Switzerland, highlighting how, after six months of therapy, the patients showed a significant improvement that tended to be maximized after two years of treatment and was confirmed in follow-up [40]. A systematic review of randomized studies using body psychotherapy in a clinical setting revealed a moderate effect in terms of efficacy on psychopathology and psychological distress [39].

## 3. Embodied Trauma and Bioenergetic Therapy

Embodied trauma is represented very well in the conditions of violence suffered. The verbal aspect loses value within a homeostatic process, which instead flows into the somatic system [26,41,42]. This can happen in terms of both awareness and habituation, in post-traumatic stress disorder (PTSD) and also in situations of chronic violence (e.g., domestic violence) [43]. To give meaning to one’s existence, the individual designs, produces, and creates satisfying relationships. Trauma interrupts this process by preventing the individual from staying in the present, so the outside world appears distant, inaccessible, or intrusive. Traumatic experiences, therefore, leave traces in the mind and emotions as well as in the biology and immune system.

The autonomic nervous system, strongly involved in endogenous responses to stress and trauma, is regulated by integrated brainstem reflexes and cranial nerves. It activates the facial, ear, and throat muscles, allowing us to scream, grimace in fear, and listen to incoming help responses [44,45,46]. If no one responds to the request or the individual does not have the necessary time to ask because the danger is imminent, the body returns to a less relational but more primitive mode of survival, which constitutes the second level: the fight or flight responses (i.e., sympathetic nervous system) [47]. In this, the limbic system comes into play, which activates the sympathetic nervous system by mobilizing the muscles, heart, and lungs to allow for a quick release. If there is no escape, the third level comes into play: freezing or collapsing.

When a person suffers a trauma, they develop somatic symptoms that are a bodily expression of psychological problems; consequently, the body also suffers. This trauma, according to Caizzi, is embodied in the subsymbolic modality that involves the affective, somatic, sensory and motor modalities of mental processing [48]. It is important to note that for many trauma survivors, the body can become a source of pain, intrusion, and shame. Therefore, survivors can often feel disconnected from their bodies. It has been established that exposure to the threat of trauma stimulates the autonomic nervous system, with consequent sympathetic hyper-excitation and parasympathetic hypo-excitation states that accompany survival responses (fight, flight, submission, and freezing). The relevant literature highlights how trauma produces a recalibration of the brain’s alarm system, an increase in stress hormones, and alterations in the system responsible for discriminating irrelevant information. Trauma compromises the area of the brain that transmits the physical, corporeal perception of being alive. These changes account for why traumatized individuals are hypervigilating the threat at the expense of being spontaneously involved in their lives.

The human body acts as a “warehouse” for everything an individual experiences during life; this leads to repressed and trapped emotions within multiple parts of the body that cause muscle tension. One of the causes of muscle tension can be precisely trauma: when an individual lives a traumatic experience and does not face it consciously, they are afflicted with fear, a stress that, if chronic, can define the structure of PTSD. All this accumulates within the body, resulting in muscle tension, which contributes to numerous other diseases, such as chronic pain, fibromyalgia, gastrointestinal disorders, and hypertension [49,50,51].

In this case, we speak of “body muscle memory”, which refers to the traces that the past has imprinted on an individual’s body. There are different types of body memory: memory constituted by the baseline tone of the muscles, which can be altered due to traumatic experiences; memory constituted by habitual postures that represent a limitation in the flexibility of relationships and an unconscious source of malaise and discomfort; memory of movements that, if repeated several times in the same circumstances, become characteristic of a person; and finally, memory of the breath, which, if altered, weighs on the well-being of the subject [52,53,54]. Therefore, trauma results in a fundamental transformation of how the mind and brain organize perceptions, changing the way we think, our actual ability to think, and our physical and hormonal responses. At the same time, the mind needs to be supported in restructuring the meaning processes associated with trauma; the body needs to learn that the danger has passed and can return to live in the present reality.

Therefore, since trauma affects the mind and body, one of the many treatment approaches used is sensorimotor psychotherapy; those who have suffered a trauma lose the “somatic connection” with the present reality: at the body level, the responses are experienced as past events that occur “again and again”; therefore, sensorimotor therapy allows evaluation of and intervention with the trauma symptoms, dealing directly with the body and then accessing the most primitive, automatic, and involuntary brain functions that underlie traumatic and post-traumatic responses [55].

The individual learns to regulate the hyperactivation and physical insensitivity (bodily, cognitive, and emotional experiences) associated with the trauma in a conscious way, being encouraged to observe and carefully describe the interaction between thoughts, emotions, physical sensations, and bodily movements that occur in the here and now. The person can thus discover that the reactions elicited in everyday life are fed by the cognitive patterns linked to the trauma, which activate the defensive responses aimed at survival.

It should be noted that memories of trauma are prone to distortion; in fact, most people remember more trauma than they actually experienced. This distortion is due to a failure to monitor their sources: following an experience, intentional and involuntary memories can introduce new details that are assimilated into the person’s memory of the event.

To heal from trauma, it would be advisable to start by improving the survivor’s awareness and knowledge of the body’s responses to trauma, with the ultimate goal of localizing a sense of security within the body. This allows the survivor to operate with a deep self-awareness rather than classic conditioning [56].

Trauma victims cannot heal until they become familiar with their bodily sensations. Being scared means having the body always on alert. To change, people need to become aware of their feelings and how the body interacts with the world around it. Body self-awareness is the first step to getting rid of the past. The therapeutic process, therefore, starts primarily from the physical sensations underlying the emotions, such as pressure, heat, muscle tension, tingling, and a sense of emptiness. Observation of bodily changes, such as chest tightness or stomach cramps, when verbalizing negative events from which the person declares not to be disturbed can give space to what is defined as somatic re-enactment or somatic responses to flashbacks related to the unprocessed event. The mind therefore needs to be re-educated to feel physical sensations, and the body needs to be helped to tolerate and enjoy contact well-being. Psychotherapy can therefore act as an intermediary to guide the individual towards a better perception of themself (bodily, emotional, and cognitive) and of the context in which they live. In doing so, psychotherapy will contribute to the psychophysical health of the individual.

Furthermore, healing can be favored by the mirror neuron system, which, due to its plasticity and immediacy, can contribute to new positive experiences that promote the formation of new adaptive, implicit procedural models. This activation can also reside in the prefrontal cortex and is part of a complex neural network that includes afferent and efferent connections to the limbic system, particularly the amygdala and the premotor and motor cortex [57,58].

Recent studies have suggested that MNS-based therapies provide a non-invasive approach to treating emotional disorders because they can modulate them through the mechanism of empathy [58,59,60,61].

## 4. Olfactory Memory and Bioenergetics: A Model of the Embodied Response Connected to Trauma

Studying the mechanisms of olfactory memory can provide information on the mechanisms of human emotions that can also be applied to the mechanisms of mental disorders involving pathological emotional processing. PTSD is of particular interest because one of its psychobiological mechanisms consists of the failure of the extinction of emotional or traumatic reminders conditioned by fear [45,62].

Olfactory memory is particularly relevant because it is closely linked to intense emotions and is distinguished from other types of memories, such as auditory and visual memory, in that it does not automatically generate verbal and/or visual representations but is, by choice, the most embodied one and evokes affective states and significant episodes. In contrast to the extensive empirical literature on the cognitive processing of verbal and visual memory, few studies have examined the role of smell in emotional or traumatic memories and in trauma-related disorders. The proximal space that the bioenergetic therapist establishes in the session and the use of the body allow, in this case, wider variation and modulation of the olfactory aspect, and also in connection with the gut–brain axis. Within this digression, Gerda Boyesen was one of the first bioenergetic psychoanalysts focused on the interoceptive system and able to use gut–brain feedback [63,64].

An information processing approach commonly includes auditory and visual stimuli to assess perception. Although these involve processes similar to those seen in olfactory memory (processing, encoding, consolidation, and retrieval), olfaction is rarely used to investigate them [65]. This is interesting because it has long been known that adding a salient olfactory cue during learning and retrieval facilitates memory recall [66].

## 5. Olfactory Stimuli, Body, and Memory in Trauma

Intrusive re-experiencing, a core symptom of PTSD, has traditionally been described through the mechanism of classical fear conditioning [45,67]. In a study of 100 refugees attending a psychiatric clinic, 45% reported having panic attacks triggered by olfactory stimuli; among these, 58% experienced at least one intrusive memory due to the smell [68].

Patients with PTSD respond intensely to trauma-related danger signals, even in objectively safe environments, and are apparently incapable of adapting their responses based on the contextual signals present. A Vietnam veteran said he was fine until, many years after the war, a Vietnamese restaurant opened near his home: he could not stand the aroma of a typical sauce used to season fish, which he had smelled several times in Vietnam. The former soldier stated that the smell brought back memories of his war experiences, making him feel discomfort and the strange sensation of being torn and covered in blood [69].

Some studies have also demonstrated increased odor detection and sympathetic arousal, including skin conductance and heart rate, in correspondence with odors related to fear, trait anxiety, or mood disorder anxiety [70]. Smells can trigger ancient and emotional memories, including memories of traumatic experiences, because the anatomy of the olfactory system involves activation of the same brain structures that support emotion processing (limbic system and medial temporal lobe circuits) and declarative memory [71].

To understand the cerebral processes involved in PTSD, it is also important to study the functioning of the breath and the olfactory system. Some studies in the literature have examined the effect of burning odors on war veterans, and it has been demonstrated that heart rate increases as a function of the negative valence (i.e., the unpleasantness), causing an increase in the stress and anguish suffered by soldiers with PTSD [72]. Sensitivity to burning odors is also correlated with the time that has passed since the trauma: in contrast to the general symptoms of PTSD, which tend to become less severe as time passes, the symptoms triggered by these types of odors can intensify [73].

Additionally, a study conducted by Croy and colleagues measured odor-related chemosensory potentials in adults who had experienced childhood maltreatment, reporting a faster processing of unpleasant odors and the increased severity of symptoms of the disorder. Grounding—positioning the subject in a posture in which they should be balanced—allows and amplifies the possibility of breathing [74]. In turn, breathing enables an increase in attentional and sensory capabilities, both physical and chemoreceptive, and also enhances aspects connected to interoception and memory retrieval [71,75].

## 6. Conclusions

This review explores the intersection of TRD, PTSD, and body psychotherapy, mainly focusing on the concept of embodied simulation and its implications for therapeutic intervention in bioenergetic psychotherapy. It emphasizes that trauma affects both the mind and body, necessitating integrated and embodied therapeutic interventions.

The concept of embodied memory, rooted in sensorimotor experiences, is highlighted as a critical aspect of understanding how trauma affects cognitive functions and emotional responses. Recognizing the role of bodily sensations and reactions is crucial in addressing trauma-related symptoms. The efficacy of bioenergetic psychotherapeutic treatment in addressing TRS suggests significant improvements in psychopathology and psychological distress over time.

Moreover, the paper discusses the significance of olfactory memory in trauma processing and symptomatology. It suggests that olfactory stimuli can trigger emotional memories, emphasizing the need to incorporate olfactory experiences into therapeutic interventions.

These aspects have practical implications for clinical practice, including incorporating somatic interventions, breathwork, and sensory experiences into trauma therapy sessions. We also emphasize the need for therapists to be attuned to clients’ verbal and nonverbal cues, including bodily sensations and emotional responses.

Future research could focus on exploring the neural mechanisms underlying embodied memory, investigating the specific effects of olfactory stimuli on trauma processing, and further evaluating the long-term effectiveness of bioenergetic therapy for trauma-related disorders.

Furthermore, this review highlights the importance of adopting an integrated, embodied approach to trauma therapy, incorporating insights from clinical psychology, cognitive neuroscience, psychophysiology, and bioenergetic practice.
